# Design and application of a novel telemedicine system jointly driven by multinetwork integration and remote control: Practical experience from PLAGH, China

**DOI:** 10.1049/htl2.12057

**Published:** 2023-12-05

**Authors:** Ruiqing Wang, Jie Zhang, Shilin He, Huayuan Guo, Tao Li, Qin Zhong, Jun Ma, Jie Xu, Kunlun He

**Affiliations:** ^1^ Medical Big Data Research Center Chinese PLA General Hospital Beijing China; ^2^ Medical Engineering Department Chinese PLA General Hospital Beijing China; ^3^ Information Department Hainan Hospital of Chinese PLA General Hospital Beijing China; ^4^ Beijing HongYun RongTong Technology Co., Ltd Beijing China

**Keywords:** data communication, emergency management, Internet of Things, medical information systems, patient diagnosis, patient treatment, telemedicine

## Abstract

In China, several problems were common in the telemedicine systems, such as the poor network stability and difficult interconnection. A new telemedicine system jointly driven by multinetwork integration and remote control has been designed to address these problems. A multilink aggregation algorithm and an overlay network for telemedicine system (ONTMS) were developed to improve network stability, and a non‐intervention remote control method was designed for Internet of Things (IoT) devices/systems. The authors monitored the network parameters, and distributed the questionnaire to participants, for evaluating the telemedicine system and services. Under a detection bandwidth of 8 Mbps, the aggregation parameters of Unicom 4G, Telecom 4G, and China Mobile 4G were optimal, with an uplink bandwidth, delay, and packet loss ratio (PLR) of 7.93 Mbps, 58.80 ms, and 0.06%, respectively. These parameters were significantly superior to those of China Mobile 4G, the best single network (*p* < 0.001). Through the ONTMS, the mean round‐trip transporting delay from Beijing to Sanya was 76 ms, and the PLR was 0 at vast majority of time. A total of 1988 participants, including 1920 patients and 68 doctors, completed the questionnaires. More than 97% of participants felt that the audio and video transmission and remote control were fluent and convenient. 96% of patients rated the telemedicine services with scores of 4 or 5. This system has shown robust network property and excellent interaction ability, and satisfied the needs of patients and doctors.

## Graphical abstract

In China, several problems are common in the telemedicine systems, such as the poor network stability and difficult interconnection. The authors’ study aims to establish a new telemedicine system based on the practices of the Chinese People's Liberation Army PLA General Hospital (PLAGH) that is jointly driven by multinetwork integration and remote control. The telemedicine practice in PLAGH proves that this system has robust network property and excellent interaction ability which wins a high level of satisfaction between patients and doctors. In addition, it has the potential to be applied in certain special situations (disaster relief, field, cabin hospitals).

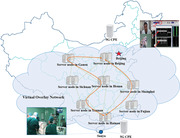

## 1 INTRODUCTION

Although there is no uniform definition of telemedicine, it mainly refers to the long‐distance transmission of text, images, and videos generated by medical institutions through communication technology, computer technology, and multimedia technology for remote clinical detection, diagnosis, and treatment [1, 2]. Telemedicine could overcome the limitations of time and space and shorten the distance between doctors and patients. China has a vast territory, however, the distributions of its population and medical resources are uneven: approximately 36% of the population lives in rural areas, while approximately 80% of medical resources are concentrated in large‐ and medium‐sized cities [3, 4]. Therefore, the implementation of telemedicine could address regional inequality in the distribution of medical resources in China, reduce the medical costs of patients, and improve the timeliness of disease monitoring, diagnosis, and treatment [5, 6].

Telemedicine systems have been booming for decades. Interactive television technology and satellite technology were mostly adopted in the initial telemedicine system, and the transmission content was mainly simple human physiological parameters [7]. The connection mode of the second‐generation system was mostly satellite and integrated services digital network (ISDN) with internet access rates of only 128 kbps and high costs [8, 9]. Since the beginning of the 21st century, through the development and popularization of telecommunications technology, telemedicine systems have been able to facilitate medical services based on various networks, such as private networks [10], the internet [11, 12], and cellular networks [13, 14]. However, there is relatively singular network access in telemedicine systems: either only a single network connection can be used or only one of multiple network connections can be manually selected, which not only wastes network resources to a certain extent but also weakens the robustness of the access network [15, 16]. In addition, assuring quality of service (QoS) for time‐sensitive services like tele‐ultrasound and telesurgery in traditional and internet protocol (IP)‐based networks is always facing lots of constraints and has made tele‐ultrasound or telesurgery impossible or possible by spending too much cost [17]. Finally, during telemedicine clinical specialists need to have access to patient examination information at all times and, if necessary, manipulate medical Internet of Things (IoT) devices/systems in real time, whereas telemedicine services are mainly based on videoconferencing, which could only provide a certain degree of interoperability (manipulation of each other's screens) [18, 19].

The People's Liberation Army PLA General Hospital (PLAGH), the largest telemedicine centre in China, is one of the first medical organizations in China carrying out telemedicine exploration [20]. This paper aims to establish a novel telemedicine system based on the PLAGH's practices that is jointly driven by multinetwork integration and remote control and conducted a feasibility study and evaluation. In addition, this paper develops a multilink aggregation algorithm by BWV of link (MLA‐BWV) and establishes an overlay network for telemedicine system (ONTMS), to ensure that the new telemedicine system could provide corresponding end‐to‐end QoS for different services through the public network (Internet/IP network). Meanwhile, a non‐intervention remote control method is designed to freely control medical IoT devices and systems, achieving the sharing and cooperation of people, data, and devices at the cross‐network and cross‐domain level.

## 2 MATERIALS AND METHODS

### 2.1 System architecture design

**FIGURE 1:**
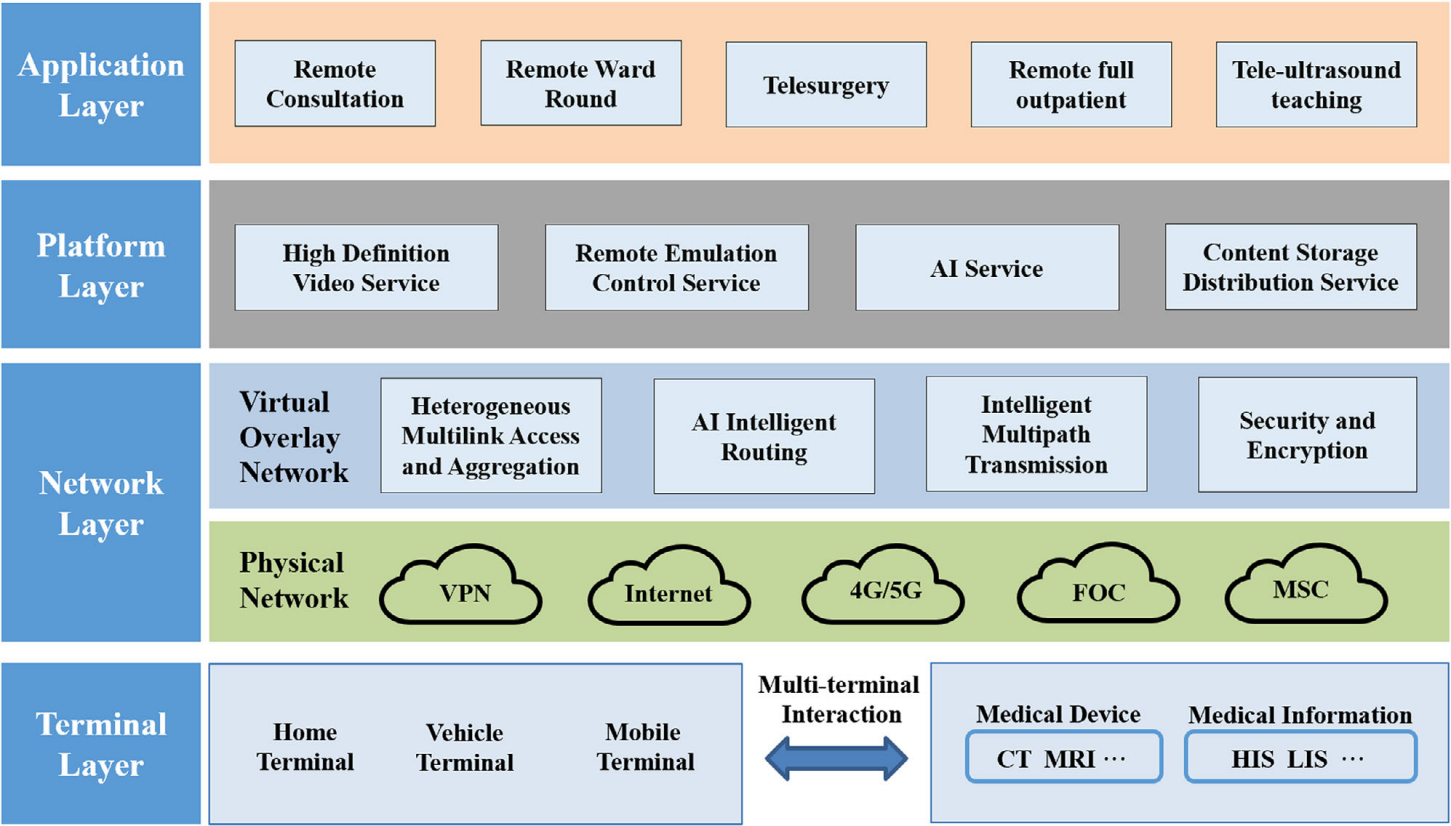
Overall framework and structure of the telemedicine system. AI, artificial intelligence; FOC, fibre‐optic communication; HIS, hospital information system; LIS, laboratory information system; MSC, mobile satellite communication; VPN, virtual private network.

The telemedicine system adopted the ‘cloud + terminal’ technology architecture, a specific implementation of cloud computing. The overall architecture model of the system was constructed through the multilayer design of the terminal layer, network layer, platform layer, and application layer (Figure 1). This system was able to connect people, data, and devices as a bus did. Here, people referred to doctors and experts, data referred to the data from various medical information systems (Hospital Information System (HIS), Laboratory Information System (LIS) etc.), and devices referred to various medical devices (CT, MRI etc.).

#### Terminal layer

This layer was the sender and receiver of network information. With the help of various terminal devices, this layer could interface with doctors’ workstations and medical IoT devices to achieve data collection and multiterminal interaction.

#### Network layer

To be compatible with various physical networks of different medical institutions and to ensure stable data transmission, we deployed server nodes at different physical network levels and used software to define a virtual overlay network with multipath transmission, intelligent routing, and secure encryption.

#### Platform layer

This layer adopted a distributed architecture to support the free access and expansion of various services, such as high‐definition video service, remote control service, AI service etc.

#### Application layer

In view of the actual needs of telemedicine participants, this layer supported diversified telemedicine services and remote collaboration across multiple regions, hospital districts, and disciplines.

### 2.2 Multinetwork integration

**FIGURE 2:**
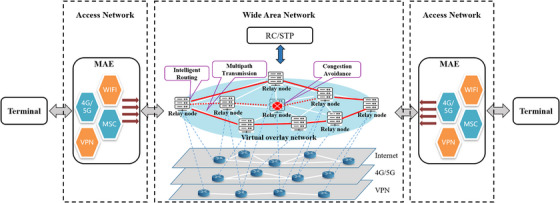
Multinetwork integration diagram. The system terminal connected to the edge relay node of the virtual overlay network through MAE, and the data was transmitted to the target terminal through multiple physical networks and multiple paths under the command of the RC and STP server. MAE, multilink aggregation equipment; RC, router centre; STP, signal transfer point.

In this paper, the concept of multinetwork integration was adopted to ensure the stability of network transmission between different telemedicine terminals, in which multilink aggregation technology was used in the access network and overlay network technology was used in the wide‐area network (WAN) (Figure 2).

#### 2.2.1 MLA‐BWV

To improve the robustness of the access network, a multilink aggregation algorithm based on the bandwidth volume (BWV) of link was developed [21]. The specific implementation algorithm was as follows:

1. Estimate the bandwidth of each link by the improved transmission control protocol (TCP) congestion window algorithm, and obtain the parameters such as window size (WS), round‐trip time (RTT), and packet loss ratio (PLR) at the same time;
2. Calculate the BWV of each link according to formula (1);
(1) $$\mathrm{BWV}=\left(\mathrm{WS}\times 1000\right)/\mathrm{RTT}\times \left(1-\mathrm{PLR}\right)$$
3. Choose a link with a larger BWV to send the current data packet and then reduce the BMV of the chosen link.

To ensure real‐time transmission, a user datagram protocol (UDP) was used to send data. With the help of the MAEs loaded with this algorithm, our system could aggregate the bandwidth of each link in the access network to meet telemedicine scenarios with high bandwidth requirements.

#### 2.2.2 ONTMS

We established a self‐discovery, self‐organizing, and telemedicine‐oriented virtual overlay network by distributing relay nodes, which was a specific cloud server for data relay and above the multiple physical networks. When a system terminal in a certain area joins the overlay network, it requests a relay node from the STP server. According to the network conditions of the terminal and relay node, the relay node in the same area was selected for the terminal to achieve the closest and optimal access. Multipath transmission algorithm was adopted for data transmission [22]. During the transmission process, the route centre (RC) monitors the transmission status information (PLR, delay, and jitter) of each path in real time and adaptively adjusts the load of each path according to the QoS) to achieve the optimal transmission combination. In addition, the ONTMS also configured appropriate priorities for different types of medical data, giving priority to the stable transmission of voice, control command, ROI in images, and so on under adverse network conditions.

### 2.3 Remote control

**FIGURE 3:**
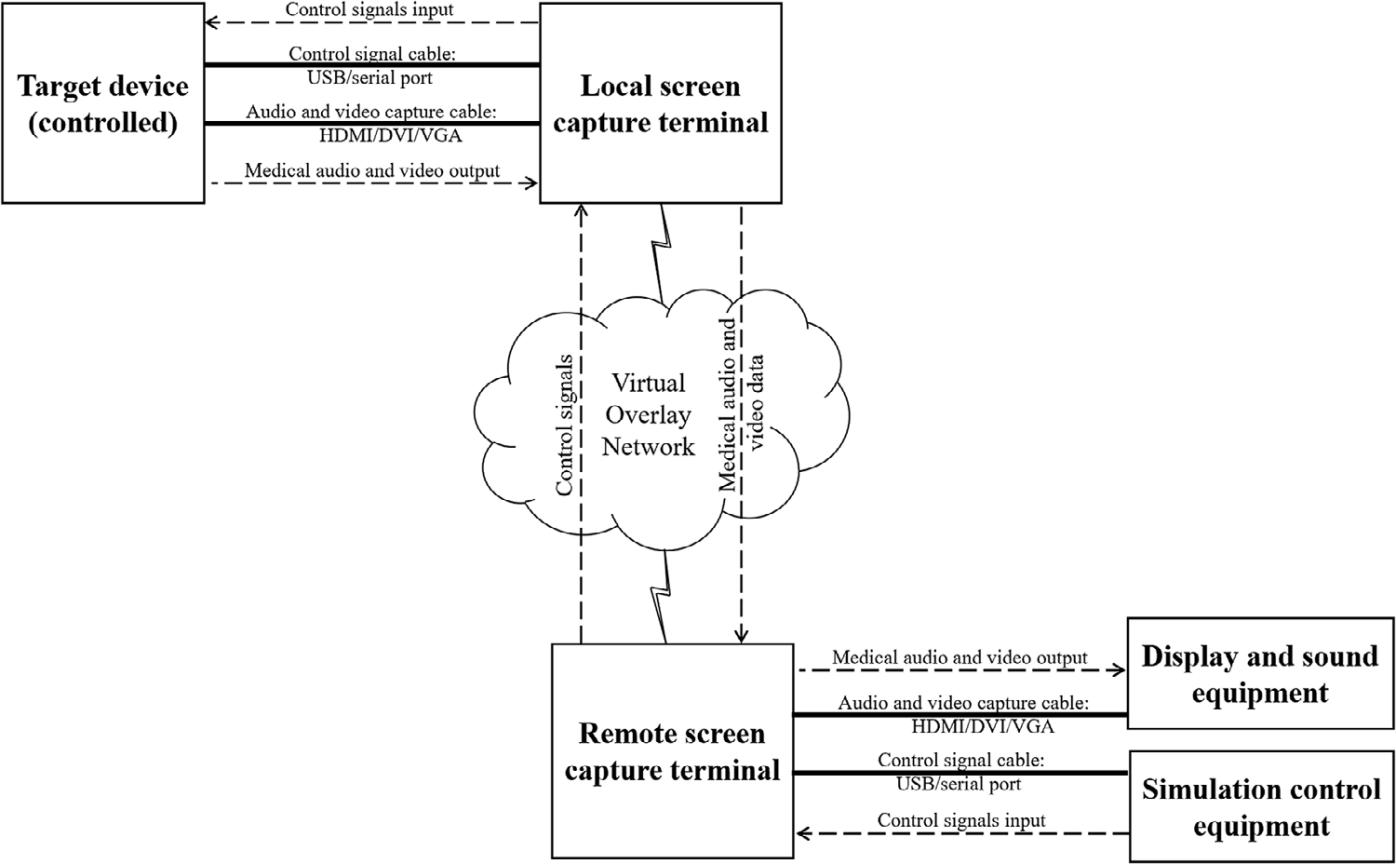
Schematic diagram of remote control.

A screen capture terminal was designed to realize remote control of the target device through screen capture and operation command simulation [23]. The local screen capture terminal was connected to the target device, the remote capture terminal was connected to the display equipment, sound equipment, and simulation control equipment, and the two terminals were able to communicate over the virtual overlay network (Figure 3). This method could also avoid controlling distant medical devices through the TCP/IP layer of the network and ensure network security while reducing the difficulty of system implementation.

#### Screen image capture

Compared with the traditional structured data acquisition through an interface, the screen image of the target device was captured through audio and video capture cables such as high definition multimedia interface (HDMI)/digital visual interface (DVI)/video graphics array (VGA) in our method, which contained information such as patient medical records and images, and then the data were presented to the remote expert in the form of video streaming to easily realize the acquisition and sharing of data.

#### Operation command simulation

Operation commands were simulated by the screen capture terminal and sent to the target device through the control signal cable, such as keyboard, mouse, or other I/O signal cables (USB/serial port) in our method. At the same time, the image of the operation result was transmitted back to the remote expert in real time to realize the synchronization of the device screen and operation commands.

### 2.4 System test method

#### 2.4.1 Test for access network

A network monitoring software was developed to detect the bandwidth, delay, and PLR of the access network. The detection steps were as follows:

- Set the detection bandwidth (Mbit/s)
- Select the nearby relay nodes
- Send data packets to the relay nodes and start testing

The calculation method of each parameter is shown in Table 1.

**TABLE 1:** Calculation method of QoS.

QoS	Method
Delay	The sender marks the sending timestamp t1 in the sent packet, and the receiver will return a response packet (carrying the sender's timestamp) after receiving it. After receiving the response packet, the sender records the reception time t2. Then, the unidirectional delay is calculated as Δ*t* = (t2−t1)/2 (unit: ms).
PLR	There is an independent packet sequence number on the end‐to‐end and each transmission segment. The receiver calculates the packet loss rate through the actual received number of packets *m* and the total number of packets *n* as PLR = (*n*−*m*)/*n* × 100%.
Traffic	The receiver calculates the traffic per second based on the number of bits *N* received within a period of *t* seconds as Traffic = *N*/(t*1000000) (unit: Mbps).

*Note*: the above parameters are calculated every second and updated every 5 s.

PLR, packet loss ratio; QoS, quality of service.

#### 2.4.2 Test for overlay network

The network monitoring software was also used to monitor the network performance parameters (delay, PLR, and traffic) between relay nodes in ONTMS during telemedicine services.

#### 2.4.3 Test for users’ satisfaction

The questionnaire was developed following the procedure used by López et al. and Yip et al. [24, 25], and expanded the equipment working condition (Additional file A). The questionnaire consisted of nine questions, which included two dichotomous questions (yes/no) and seven statements using a 5‐point Likert scale (strongly agree/very satisfied to strongly disagree/very dissatisfied).

The study was approved by the ethics committee of Hainan Hospital of Chinese PLA General Hospital (reference number: 301HNFY‐11). Written consent was obtained from each participant following an explanation of the purpose of the study.

#### 2.4.4 Statistical methods

The Kolmogorov‒Smirnov test was used to assess the normality of the data. Descriptive statistics were used to compute percentages, and means and standard deviations. Student's t test or Kruskal‒Wallis test, when appropriate, was employed to analyze the bandwidth, delay, and PLR of different access networks. SPSS V 25.0 was used for statistical analysis. A *p* value of < 0.05 was considered statistically significant.

## 3 RESULTS

The system has been successfully deployed in PLAGH's seven medical centres (located in Beijing) and Hainan Hospital (located in Sanya) at a distance of nearly 3000 km, with the functions of high‐definition video conferencing, reliable transmission network, and remote control. Thus, PLAGH has pioneered serval the world's first 5G telemedicine services, such as 5G remote brain pacemaker implantation surgery, Mako robotic remote manipulation in total hip replacement surgery, and 5G full outpatient. In addition, a series of telemedicine services, such as remote outpatient/consultation, remote ward round, telesurgery, remote full outpatient, and tele‐ultrasound teaching, have been routinely carried out in PLAGH, serving over 3000 patients, training approximately 60,000 medical staff in total and covering 15 clinical departments, such as neurosurgery, radiology, ultrasound etc. (Table 2).

**TABLE 2:** Remote service information statistics of telemedicine collaborative system from PLAGH.

Service	Cases number	Department
Remote consultation	3367	Neurosurgery
Remote ward round	260	Gastroenterology, Neurosurgery, Neurology, Radiology, Urology, Cardiology, Otolaryngology, Ophthalmology, Nephrology, General Surgery
Telesurgery	147	Neurosurgery
Remote full outpatient	124	Dermatology, Tropical Medicine, Orthopedics, Obstetrics, and Gynecology
Tele‐ultrasound teaching	60,000	Ultrasonography

PLAGH, People's Liberation Army PLA General Hospital.

### 3.1 Detection results of access network

This study detected the network performance of system terminals located at the No. 1 Medical Centre of PLAGH (No. 1 MC) connected to nearby relay nodes. The access networks included China Mobile 4G, Telecom 4G, and Unicom 4G, which set 8 Mbps as the detection bandwidth. The measurements were averaged 10 times in each condition, and the results (uplink) were shown in Table 3. When the single network was adopted, the network performance of China Mobile 4G was the best around three networks, with an uplink bandwidth, delay, and PLR of 4.09 Mbps, 276.20 ms, and 45.38%, respectively. When the above three networks were aggregated, the network property was significantly superior to that of China Mobile 4G (*p* < 0.001): bandwidth was almost equal to detection bandwidth, delay was 58.80 ms, and PLR was almost zero.

**TABLE 3:** Network parameters of the access network (uplink).

	Bandwidth (Mbps)	Delay (ms)	Packet loss ratio (%)
Telecom 4G	1.16 ± 0.18	1247.20 ± 200.29	82.14 ± 3.01
Unicom 4G	3.00 ± 0.56	1062.40 ± 348.64	55.89 ± 6.88
China Mobile 4G	4.09 ± 0.18	276.20 ± 16.93	45.38 ± 3.06
All	7.93 ± 0.40^**^	58.80 ± 3.96^**^	0.06 ± 0.12^**^

*Note*: **: *p* < 0.001, compared with China Mobile 4G.

### 3.2 Monitoring results of overlay network

**FIGURE 4:**
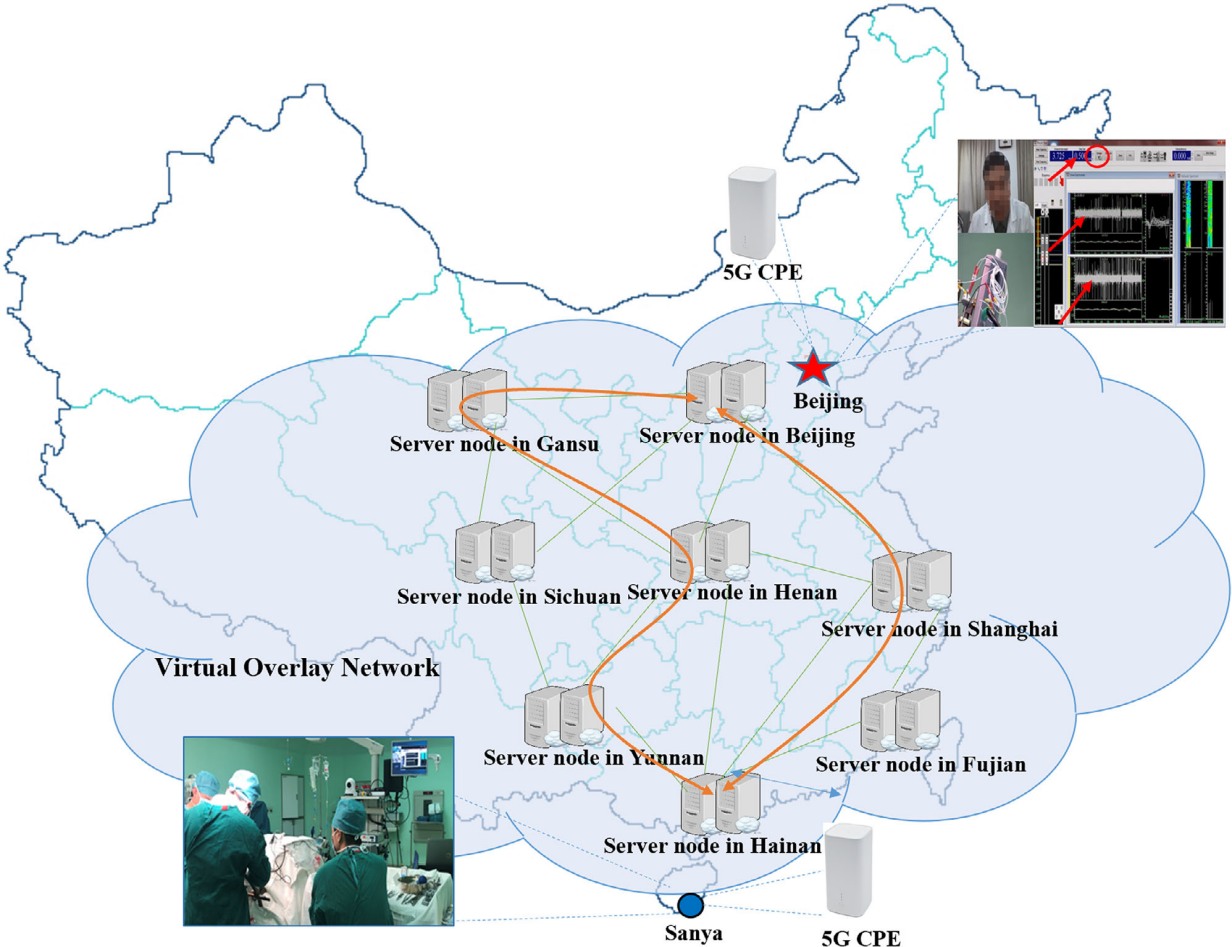
Telesurgery network connection solution. Both parties were connected to nearby server nodes via 5G customer premise equipment (CPE), and a reasonable path was selected for data transmission by intelligent algorithms. Arrows and circles were drawn for multiparty cooperative labelling.

Taking 5G remote brain pacemaker surgery as an example, the clinical experts located at the No. 1 MC remotely operated the Alpha Omega neurophysiology system located at Hainan Hospital and achieved the precise positioning of microelectrodes according to the Microelectrode Recording Signal (MERS) transmitted back in real time. While controlling the device, the remote experts could also annotate what is displayed on the device, and the annotation type could be arrows, circles, mosaics etc. (Figure 4).

**FIGURE 5:**
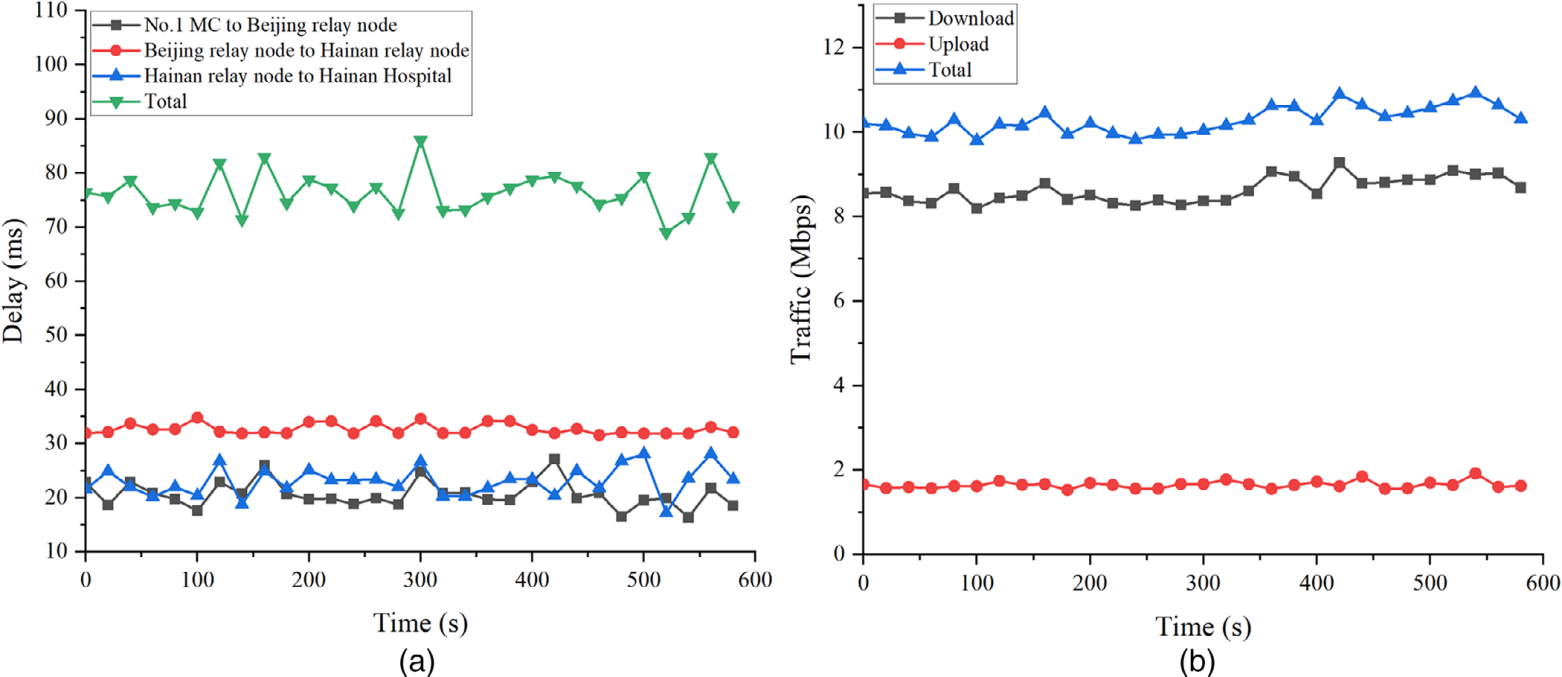
Monitoring curves for average network performance parameters from 147 telesurgeries. (a) Refers to average round‐trip transporting delay curves from No. 1 MC to Hainan Hospital; (b) Refers to average traffic curves of No. 1 MC.

Figure 5 shows the average network parameters from 147 telesurgeries. The average delays from No. 1 MC to the Beijing relay node, the Beijing relay node to the Hainan relay node, and the Hainan relay node to Hainan Hospital were 21, 33, and 23 ms, respectively, and the total was 76 ms. The sending and receiving traffic of No. 1 MC was generally stable, and the overall traffic was approximately 10 Mbps. We omitted the results of the PLR in Figure 5 because they were 0 at the vast majority of times.

### 3.3 Users’ satisfaction results

From July 2020 to March 2023, we continuously included 1988 participants (1920 remote outpatients and 68 medical workers), and distributed questionnaires after obtaining informed consent. The patients answered the first six questions and the doctors answered the last five questions. Their answers are summarized in Table 4. In terms of system performance, more than 97% of doctors and patients participating in telemedicine did not feel the delay in communication and video transmission, and more than 96% of doctors felt that remote control and remote labelling were convenient. 96% of patients were satisfied with telemedicine, 92% of patients found that telemedicine sessions were as good as ordinary face‐to‐face visit, and 94% of patients would choose telemedicine again. 96% of patients believed that telemedicine reduced medical and time costs.

**TABLE 4:** User evaluation results.

Statements		Number (%)
How satisfied were you with this telemedicine?	Satisfied and above	1843 (96)
Do you think your telemedicine session was as good as ordinary face‐to‐face visit?	Good and above	1766 (92)
Would you choose telemedicine again?	Yes	1804 (94)
Do you think telemedicine reduces your medical costs and time costs?	Yes	1843 (96)
Smoothness of the video picture	Smooth and above	1948 (98)
Synchronization of audio and video	Synchronous and above	1928 (97)
How easy was it to operate the system?	Easy and above	67 (99)
How convenient was it to remotely control medical devices?	Convenient and above	65 (96)
How convenient was it to remotely label?	Convenient and above	66 (97)

Values shown are number (percentage) of people who answer 4, 5, or yes.

## 4 DISCUSSION

In this study, the concept, design, and application of a telemedicine system jointly driven by multinetwork integration and remote control are proposed to address the challenges of the poor robustness of access networks and WANs and the limited collaboration between people, data, and devices. Unlike the deficiencies of traditional telemedicine systems, such as fixed locations, expensive private networks, and relatively singular services [10, 26, 27], our system is based on overlay network for telemedicine (ONTM), and deployed with the architecture of ‘cloud + end’. Furthermore, we propose a general, non‐intrusive remote control technology [7], which controls remote equipment and systems through screen capture and simulation control, avoiding traditional interface development and control software implantation. This system has been successfully deployed in PLAGH and has cumulatively facilitated more than 100 remote surgeries, over 3000 comprehensive remote diagnoses and more than 150 sessions of tele‐education; it has been shown to be feasible, effective, and efficient in video conferencing, data transmission, and remote control, enabling direct interactions among healthcare providers and patients.

MLA‐BWV was developed and was loaded to MAE, which could support common network video streaming protocols (TS, RTP, RTMP, etc.) and aggregate the bandwidth of the available network when necessary. Several studies [16, 28] have pointed out that the network connections in China's telemedicine system are mostly virtual private network (VPN) (a special telemedicine network constructed in China based on wired network), internet, and cellular network. The 4G network test results also showed that the network performance after aggregation was much better than that of the single network of the traditional telemedicine system. Consistent with the results of this study, in a research of wireless real‐time video transmission system [29], the transmission bandwidth is significantly improved after the aggregation of three China Mobile 4G SIM cards. Thus, the multilink aggregation technology is able to ensure the robustness of the access network to a certain extent.

Regarding telesurgery, several attempts have also been made worldwide since the Lindbergh operation in 2001 [26] (Table 5). In 2008, Nguan et al. conducted a trial of da Vinci telesurgery via a VPNe network with an average network delay of 66 ms and a maximum network fluctuation of 5 ms [33]. Zheng et al. performed four laparoscopic telesurgeries in 2020, with a 5G wireless network with a mean round‐trip transporting delay of 114 ms and a 1.20% data PLR [30]. Yang et al. in 2022 performed an ultra‐remote radical cystectomy (network communication distance of nearly 3000 km) on patient diagnosed with T2N0M0 stage bladder cancer through a 5G network with a mean round‐trip transporting delay of 104 ms and a 0% data PLR [31]. In 2022, Zhang reported that their team performed Unilateral nephrectomy in 5G wireless network and deterministic networking (DetNet) mode with an average network delay of 27 ms and a 0% data PLR [32]. Under the regulation of ONTMS, electrophysiological signals and high‐definition video during telesurgery can be transported smoothly, with an average round‐trip transporting delay of about 76 ms and a PLR of 0. In contrast, the telesurgery system presented in the literature [30, 31] uses a universal 5G network, which has a greater network transmission delay than that of this system, even though it is being used by the network operator trying its best to guarantee the quality of service during the telesurgery. Furthermore, the telesurgery system introduced in the literature [32, 33] adopts DetNet and VPNe networks, but DetNet is usually more costly to build and maintain; the VPNe network depends on the public Internet stability, which may affect the availability of the VPNe network if the Internet is unstable or fails. The delay is within the 200 ms delay range recommended in the literature [34, 35] for remote surgical operations and even shorter than the visual evoked potential time (P100 wave with an average peak latency of 97.73 ± 3.55 ms) [36]. This application not only verifies the stability and reliability of the network transmission and remote control of this system but also accumulates the experience for the clinical application of remote collaboration.

**TABLE 5:** Comparison of network performance of different telemedicine systems.

Telemedicine system	Network type	Distance (km)	Delay (ms)	PLR (%)
PLAGH	ONTMS	2900	76	0
AHQU [30]	Universal 5G	2200	114	1.2
AHQU [31]	Universal 5G	3000	104	0
AHQU [32]	DetNet	240	27	0
VGH [33]	VPNe network	2848	66.1 ± 1.5	–

*Note*: AHQU, the Affiliated Hospital of Qingdao University; PLAGH, People's Liberation Army PLA General Hospital; PLR, packet loss ratio; VGH, Vancouver General Hospital.

The questionnaire of this study mainly comes from Telemedicine Satisfaction Questionnaire (TSQ) designed by Lo´pez et al. and Yip et al. [24, 25]. The TSQ clearly addresses the three usability factors central to telehealth (usefulness, satisfaction, and interaction quality between patient and clinician over telemedicine technology). And compared with Telehealth Usability Questionnaire (TUQ) [37], which is complex and requires a great effort to understand for patients, TSQ is more suitable for the study setting of our paper. In our study, participants were highly satisfied with the telemedicine system and services. We found that more than 96% of respondents were satisfied with their telemedicine, with a score of 4 or 5 on a five‐point Likert scale. In a study from the Netherlands [38], participants were reasonably satisfied with videoconferencing; on average, they gave the service a rating of 7.5 (on a scale from 1 to 10). While 54% of Bangladesh [10] patients thought that the telemedicine session was as good as an ordinary face‐to‐face visit, 92% of China's patients thought that their session was as good as a face‐to‐face visit. Furthermore, possibly due to the increase in patients’ acceptance of telemedicine and improvements in technology (audio and video, image quality, remote control etc.), 94% of patients would choose telemedicine again.

It is worth noting that distributed services and clusters were used to conveniently expand the system processing capacity, and a variety of telemedicine services suitable for the needs of medical institutions at different levels were established and expanded, which can be extended to cell phone apps at a later stage through reserved mobile access interfaces.

Several limitations with this study must be acknowledged. First, this study only included patients in 301 hospitals, so whether the conclusions of this study can be extended to other hospitals and regions still needs further exploration. Second, the number of doctors included in this study is relatively small, and it is still necessary to include the satisfaction data of doctors who use this system in multi‐centres in the future. Third, this study was carried out during the period of COVID‐19, and the application value and effectiveness of this system in non‐epidemic period need to be further explored.

## 5 CONCLUSIONS

The practice in PLAGH proved that the new telemedicine system was feasible and reliable. Relying on the ‘cloud + terminal’ technical framework and the technical support of multinetwork integration and remote control, not only could it be conveniently and quickly deployed in various medical centres, but it also has stable and reliable real‐time transmission, remote control, and interaction functions, which effectively improves the implementation effect, service quality, and application scope of telemedicine. In addition, a high level of satisfaction was demonstrated in patients and doctors with telemedicine system performance as well as service quality in PLAGH. The design and application of the telemedicine system has potential values and inspirations for addressing the uneven distribution of medical resources, reducing medical costs, providing more accurate telemedicine services, and carrying out related services in some special scenarios.

## AUTHOR CONTRIBUTIONS

**Ruiqing Wang**: Software; writing—original draft. **Jie Zhang**: Writing—original draft. **Shilin He**: Writing—review & editing. **Huayuan Guo**: Writing—review & editing. **Tao Li**: Writing—review & editing. **Qin Zhong**: Formal analysis. **Jun Ma**: Writing—review & editing. **Jie Xu**: Software. **Kunlun He**: Methodology; project administration.

## CONFLICTS OF INTEREST STATEMENT

The authors declare no conflict of interest.

## Data Availability

The datasets generated or analysed during the current study are available from the corresponding author on reasonable request.
